# A Warning from "Bart.'s"

**Published:** 1913-07-19

**Authors:** Thomas Hayes

**Affiliations:** Clerk to the Governors St. Bartholomew's Hospital.


### The Editor's Letter-Box.

### A Warning from "Bart.'s."

*To the Editor of* The Hospital.

Sir,—May I ask your aid in bringing to the notice of the public the fact that certain persons who represent themselves as agents of the Helping Hand Legal Assistance Syndicate (late Red Cross) are obtaining money by an improper use of the name of this hospital? These persons are in the habit of calling at houses, particularly in the poorer districts, and presenting a circular issued by the above-named Society to the effect that the Syndicate are subscribers to St. Bartholomew's Hospital. They then sell tickets at 3d. each, which they state will entitle the holder to treatment at this hospital.

This statement is quite untrue, and it is not a fact that the Helping Hand Legal Assistance Syndicate are or ever have been subscribers to this hospital. I have in my possession a letter from the Secretary undertaking to withdraw the circular above referred to; but, as I find it is still in use, I desire to make it quite clear that the persons who represent themselves as the agents of the Syndicate have no connection of any kind with this hospital.—I am, Sir, your obedient servant,

Thomas Hayes,

St. Bartholomew's Hospital. Clerk to the Governors.

